# A Quantitative Model of Human Jejunal Smooth Muscle Cell Electrophysiology

**DOI:** 10.1371/journal.pone.0042385

**Published:** 2012-08-17

**Authors:** Yong Cheng Poh, Alberto Corrias, Nicholas Cheng, Martin Lindsay Buist

**Affiliations:** 1 Department of Bioengineering, National University of Singapore, Singapore, Singapore; 2 NUS Graduate School for Integrative Sciences and Engineering, National University of Singapore, Centre for Life Sciences (CeLS), Singapore, Singapore; University of Zurich, Switzerland

## Abstract

Recently, a number of ion channel mutations have been identified in the smooth muscle cells of the human jejunum. Although these are potentially significant in understanding diseases that are currently of unknown etiology, no suitable computational cell model exists to evaluate the effects of such mutations. Here, therefore, a biophysically based single cell model of human jejunal smooth muscle electrophysiology is presented. The resulting cellular description is able to reproduce experimentally recorded slow wave activity and produces realistic responses to a number of perturbations, providing a solid platform on which the causes of intestinal myopathies can be investigated.

## Introduction

Gastrointestinal (GI) motility is achieved through the interplay between smooth muscle cells (SMC) and the interstitial cells of Cajal (ICC), and is modulated by the enteric nervous system. The ICC are pacemaker cells that spontaneously generate rhythmic electrical signals, known as slow waves, that are passed to the SMC via gap junctions. The response of the SMC to the ICC slow waves is mediated by a variety of ion channels and transporters [1]–[3].

A growing wealth of experimental and clinical evidence appears to point towards a link between ion channelopathies and digestive symptoms in the small intestine [4]–[6]. Despite this, the mechanistic link between genotype and phenotype is currently missing. This may be due, in part, to the inherent difficulties in evaluating the effects of genetic abnormalities in native cells and/or in an *in vivo* experimental model. Computational models can be used to bridge this missing link. Typically, a model of cellular electrophysiology succinctly combines descriptions of the pertinent ion channels into a cohesive framework. It is then possible to alter the description of one ion channel type (e.g. simulating a mutation) and obtain a prediction of cell behaviour. This approach has been successfully employed in the cardiac field, where SCN5A mutations underlying long QT and Brugada syndromes were elucidated with suitable computational models [7], [8].

In the GI field, computational models are at a relatively nascent stage. At present the available biophysically based models describe only non-human cells, and mainly focus on the stomach [9], [10]. Earlier SMC models by Miftakhov et al [11], [12] and Skinner et al [13] describe the SMC as being self-excitatory and thus are not consistent with more recent findings where ICC provide the pacemaking activity. In the small intestine, two biophysically based and one phenomenological model of ICC exist, but there exists only a phenomenological model of intestinal SMC activity [14]–[16]. The lack of a biophysically based intestinal SMC model hinders our understanding of how intestinal motility disorders arise from abnormal ion channel behaviour. This paper, therefore, presents the first biophysically based model of human jejunal SMC (hJSMC) electrophysiology.

## Materials and Methods

### Model development

**Figure 1:**
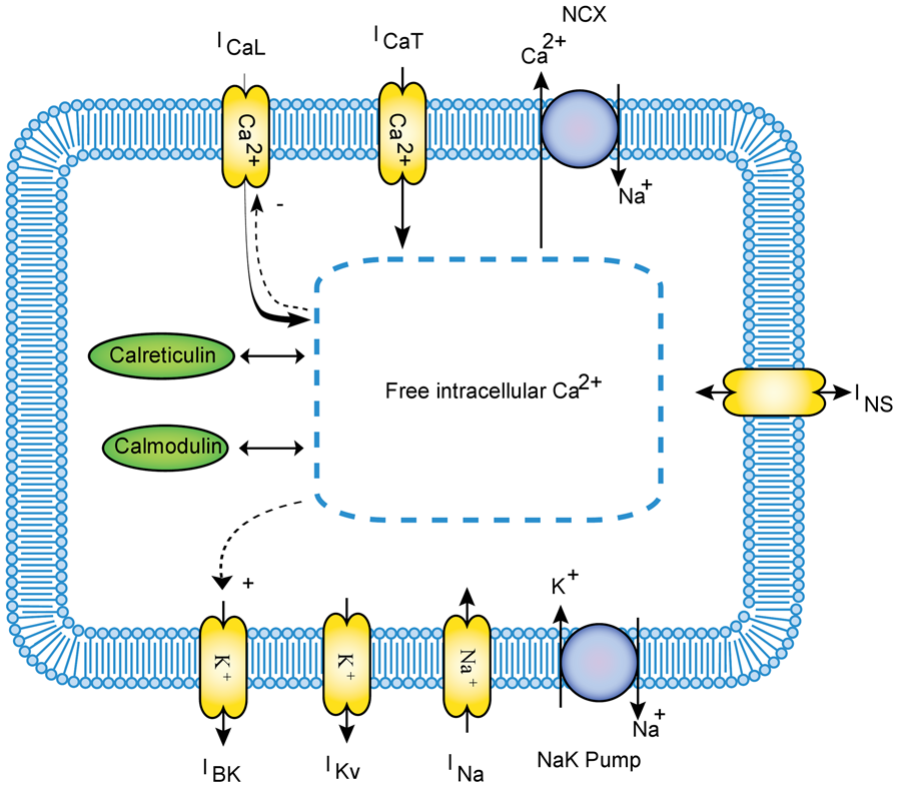
Schematic overview of the hJSMC model. It contains the ionic conductances and sub-cellular mechanisms that shape cellular electrical behaviour and intracellular Ca^2+^ dynamics.

Using the classical Hodgkin-Huxley approach that describes cellular electrophysiology as a simple circuit with conductances in parallel with a membrane capacitance [17], the governing equation for the membrane potential, 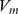, as a function of time is given by
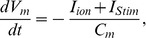
(1)where 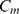 is the membrane capacitance, which was chosen to be 50 pF, within the reported range of 39 to 65 pF for human jejunal myocytes [18]–[20], and 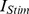 is an external stimulus current, usually provided by the ICC *in vivo*. 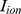 is the sum of the ionic currents crossing the membrane and is given by 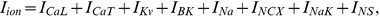
(2)where 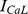 and 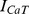 are L-type and T-type calcium (Ca^2+^) currents respectively, 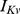 is the voltage-dependent potassium (K^+^) current, 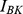 is the large conductance Ca^2+^ and voltage activated K^+^ current, 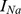 is the sodium (Na^+^) current, 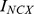 is the current due to the Na^+^-Ca^2+^ exchanger, 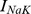 is the current generated by the Na^+^-K^+^ pump, and 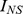 is a non-selective leakage current. Figure 1 shows a schematic of the hJSMC model.

### Ionic currents

Here, the ionic currents have been described by either a traditional Hodgkin-Huxley (HH) formalism [17] or a deterministic multi-state Markov (MM) formalism [21]. The former models ion channels as containing independent activation, and in some cases, inactivation gates while the latter models ion channels as existing in multiple closed, open and inactivated states. In either case, the current, 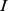, through an ion channel can be described as
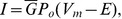
(3)where 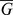 is the maximum conductance, *P_o_* is the channel open probability and 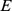 is the Nernst potential of the specific ionic species. A complete mathematical description of the hJSMC model, and the corresponding parameter values, are provided in the Supporting Information S1.

### L-type Ca^2+^ channels

**Figure 2:**
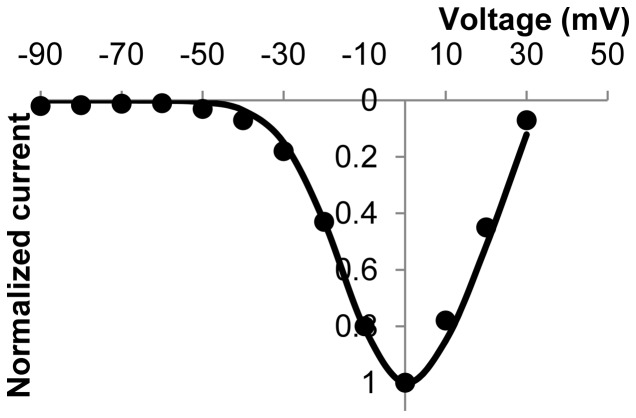
Normalized L-type calcium channels peak I–V plot for experimental (dots) and simulated data (solid line). The experimental data in this figure were adapted from Lim et al [25].

L-type Ca^2+^ channels have been identified in human jejunal smooth muscle and are considered the main pathway for Ca^2+^ entry [22], [23]. The L-type ionic current was described by 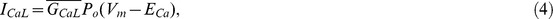
(4)where 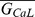 is the maximum conductance with a value of 1.44 nS. 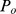 was calculated using a MM formulation with the topology proposed by Faber et al [24] (as provided in the Supporting Information S1). Lim et al characterized the kinetics of the human jejunal L-type channel by transfecting the α_1C_ and β_2_ subunits into HEK cells [25]. The rate constants governing the transitions between states have been refitted in order to replicate these data. Following the experimental conditions in Lim et al, voltage clamp simulations were conducted [25]. The presence of 2 mM EGTA in the pipette solution was replicated by switching off the Ca^2+^ dependency in the relevant state transitions. Figure 2 shows a simulated normalised current-voltage (I–V) plot along with the equivalent experimental data from Lim et al [25], while the corresponding simulated current over time results for a range of clamping voltages are shown in the Supporting Information S1.

### T-type Ca^2+^ channels

**Figure 3:**
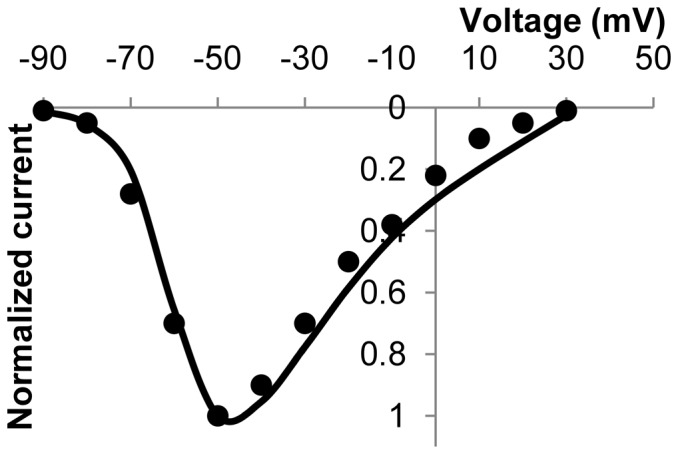
Normalized peak I–V plot for simulated (solid line) and experimental (dots) T-type Ca^2+^ currents. Experimental data in this figure are adapted from Strege et al [28].

Low-voltage activated T-type Ca^2+^ channels have been identified in intestinal SMC and ICC both by genetic expression studies [26] and by functional differentiation of two distinct Ca^2+^ currents [27]. In the human jejunum, however, the expression of T-type channels is less clear. Farrugia et al [20] observed that nifedipine completely abolished the inward currents in freshly isolated cells, suggesting that only L-type channels may be present. Nevertheless, the authors did not exclude the possibility of ‘another Ca^2+^ type with low channel density and/or low open probability’. Here, the T-type Ca^2+^ channels were included, albeit with a substantially lower whole cell maximum conductance than for the L-type Ca^2+^ channels (1.44 versus 0.0425 nS). The T-type current was modelled by
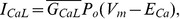
(5)where 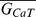 is the maximum conductance, 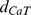 and 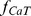 are the HH activation and inactivation gates respectively. The parameters that characterize the gates 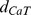 and 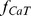 were chosen in order to replicate the kinetics that were experimentally measured in HEK cells transfected with human Ca_v_3.1 T-type Ca^2+^ channels [28]. Results of simulated voltage clamp experiments are shown in Figure 3, along with the equivalent experimental data from Strege et al [28]. Additionally, the corresponding simulated current over time results for a range of clamping voltages are shown in the Supporting Information S1.

### Voltage dependent K^+^ channels

**Figure 4:**
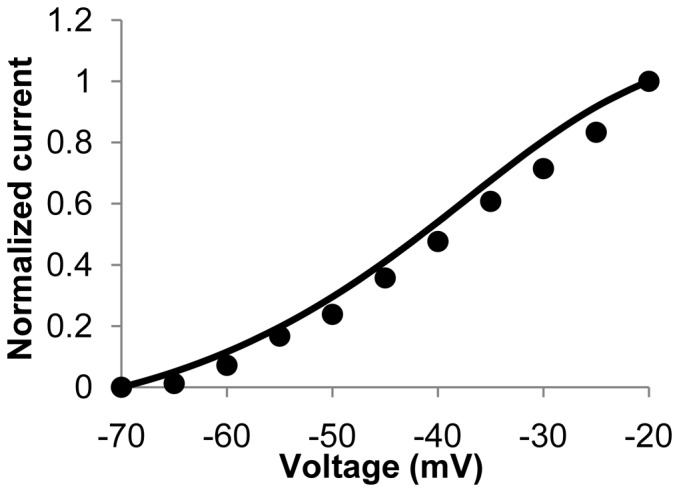
Normalized I–V plot of whole cell voltage-activated potassium currents. Simulated results (solid line) are shown together with corresponding experimental data (dots) from human jejunal myocytes [29].

Voltage gated K^+^ channels have been identified and functionally characterized in many SMC along the GI tract (see [3] for review). In freshly isolated human jejunal myocytes, a large, voltage dependent whole cell K^+^ current was measured and characterized [19], [29]. In the model, such current was described by the following equation
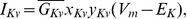
(6)where 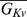 is the maximum conductance with a value of 1.0217 nS, while 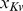 and 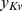 are the HH activation and inactivation gates respectively. The resulting model was able to replicate the normalized I–V plot from Farrugia et al [29] over the physiological range of membrane potentials, as shown in Figure 4. The corresponding simulated current over time results for a range of clamping voltages are shown in the Supporting Information S1.

### Large conductance Ca^2+^ and voltage activated K^+^ channels

**Figure 5:**
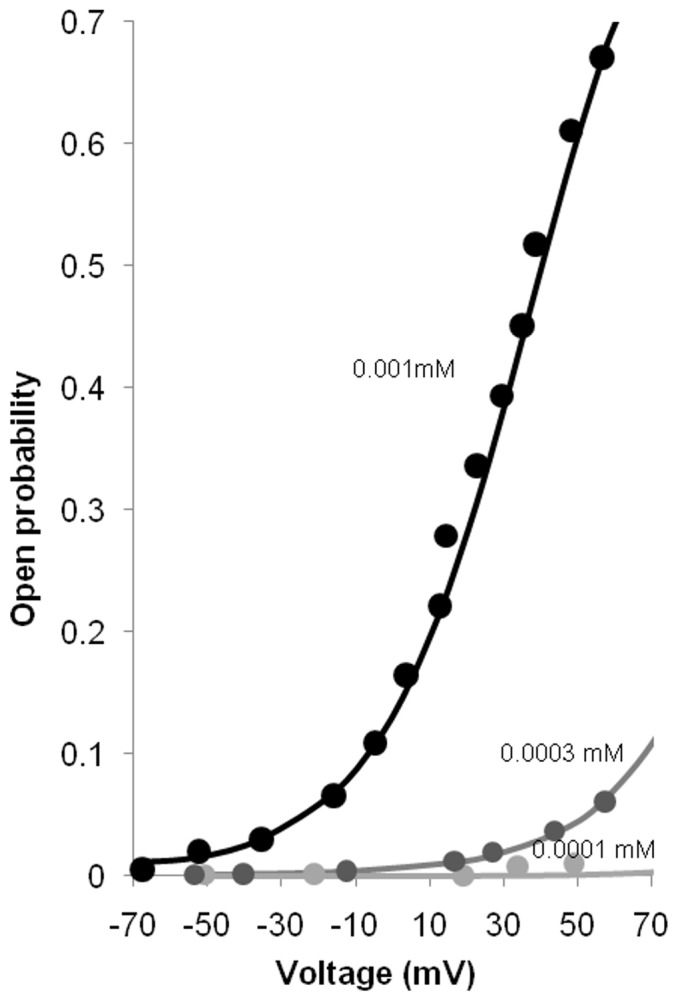
Open probability versus clamping voltage plots, across various 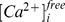 (from 100 nM to 1000 nM). Good agreement between data from experiments [35] (dots) and from the MM BK model (solid lines).

The large conductance Ca^2+^ and voltage activated K^+^ channels (BK) channels are found in the GI smooth muscles of several species [30]–[33] and their high conservation across multiple species suggests the importance of such channels in cellular electrophysiology. To describe the kinetics of BK channels, a MM description was adapted from Cox et al [34]. The model topology describes cooperative Ca^2+^ binding to each of the four alpha subunits of the BK homotetramer while transitions between a pair of closed and open states are voltage dependent (as provided in the Supporting Information S1). 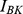 was described by
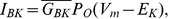
(7)where 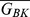 is the maximum conductance with a value of 80 nS. Due to the absence of data from human intestinal smooth muscle cells, the model's parameter values were fitted to human myometrium smooth muscle data [35]. Figure 5 shows a comparison of the BK open probability at different voltages and Ca^2+^ concentrations between simulated voltage clamp and the corresponding experimental open probabilities. The corresponding simulated open probability over time results are shown in the Supporting Information S1.

### Na^+^ channels

**Figure 6:**
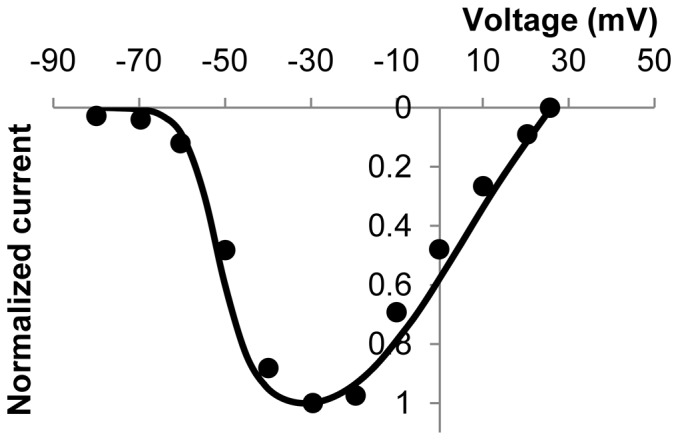
Normalized I–V plot for Na^+^ currents. Experimental data (dots) [36] is in good agreement with model data (solid line).

Na_v_1.5 channels encoded by SCN5A are expressed in the human jejunal circular SMC [36], [37]. Na_v_1.5 channels are voltage dependent and tetrodotoxin resistant, and share a near identical amino acid homology to cardiac SCN5A. 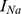 was described by
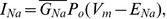
(8)where 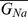 is the maximum conductance with a value of 25.1 nS. To describe Na_v_1.5 kinetics, a six-state MM model (as provided in the Supporting Information S1) was created based on experimental data from Mazzone et al's patch clamp studies on Na_v_1.5 expressed in HEK cells [5]. Figure 6 shows a normalized I–V plot from a simulated voltage clamp experiment and the corresponding human jejunal SMC data [36], while the corresponding simulated current over time results are shown in the Supporting Information S1. Further details on the Na_v_1.5 model may be found in Poh et al [38].

### Na^+^-K^+^ Pump

The Na^+^-K^+^ pump (NaK) is essential for maintaining the Na^+^ and K^+^ gradients across the cell membrane. It is almost ubiquitous in mammalian cells and has been identified in the GI tract in early studies on the guinea-pig taenia coli [39], [40]. Due to the lack of quantitative data for NaK in human jejunal smooth muscle, the formulation proposed by ten Tusscher et al was adopted and the maximum flux through the pump was selected to ensure ion homeostasis [41].

### Na^+^-Ca^2+^ exchanger

The Na^+^-Ca^2+^ exchanger (NCX) removes excess Ca^2+^ ions from the cytosol following voltage dependent Ca^2+^ entry. Its presence in the GI tract has been demonstrated through staining in murine jejunum smooth muscle [42]. As there is a lack of quantitative data about NCX activity in the human jejunum, the formulation proposed by ten Tusscher et al was adopted [41].

### Non-selective leak current

Single human jejunal smooth muscle cell studies have reported that upon inhibition of the dominant outward currents, a non-selective linear leakage current with a 0 mV reversal potential was present [19], [29]. This current was defined to include both sodium (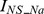) and potassium (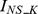) components. The total channel conductance was determined to be least 45 fold smaller than the maximum conductance of 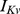.

### ICC stimulus current

**Figure 7:**
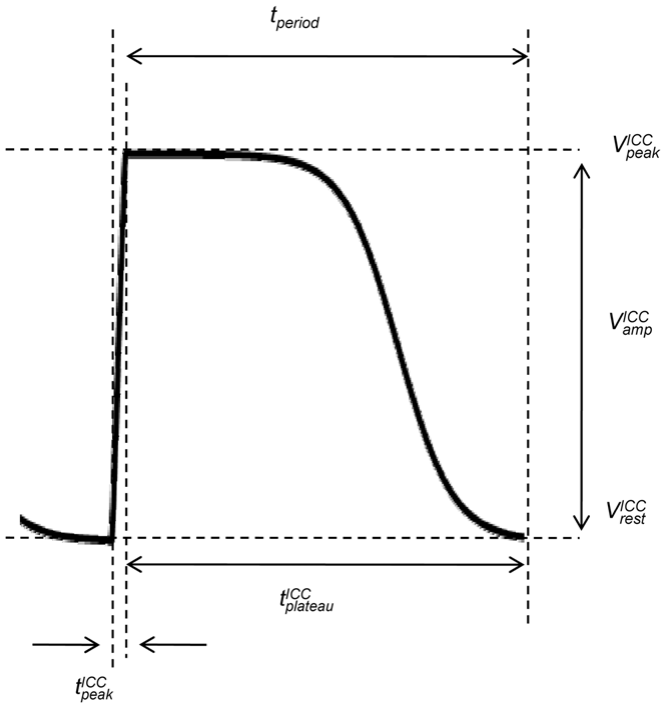
A single slow wave of ICC membrane potential 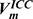. 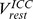
 = −57 mV, 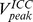 = −23.5 mV, 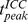 = 300 ms and 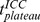 = 9700 ms. Refer to the text for further explanation.

Slow waves originate from ICC that are electrically coupled to SMC via gap junctions [43]–[46]. Hence, for an SMC, the current generated by an ICC can be thought of as an external stimulus. Here, the formulation of 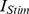 was chosen by describing the gap junctions as constant conductance between ICC and SMC,
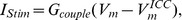
(9)where 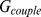 represents the coupling conductance between ICC and SMC while 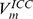 is the membrane potential of the adjacent ICC which, in the absence of a biophysically based description of a human jejunal ICC, was described phenomenologically as
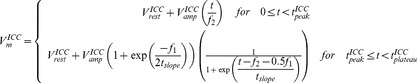
(10)Here 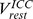 is the ICC resting membrane potential, 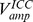 is the amplitude of the slow wave, and their sum gives the peak potential, 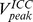. 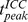 is the duration of the upstroke and 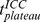 is the duration of the plateau phase, and together they sum to give the slow wave period, 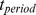. Finally, 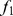, 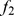 and 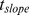 are scaling constants. The resulting 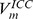 waveform is shown in Figure 7.

The frequency of slow wave activity was chosen to be 6 cycles per minute (cpm) (or 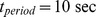) based on experimental recordings from human jejunal myocytes at 6.03±0.33 cpm [47].

### Intracellular ion concentrations

Intracellular Ca^2+^ levels are of primary importance in SMC as one of the key drivers of their contractile function. Furthermore, intracellular Ca^2+^ is known to exert a direct regulatory effect on certain classes of ion channels (e.g. L-type Ca^2+^ channels and BK channels). Ca^2+^ enters the cell through membrane Ca^2+^ channels (L-type and T-type) and is primarily extruded out of the cytoplasm by the Na^+^-Ca^2+^ exchanger. The total intracellular Ca^2+^ concentration as a function of time is given by 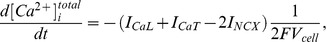
(11)where 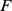 is Faraday's constant, and 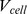 is the cell volume. Upon entering the cell, a fraction of the total Ca^2+^ concentration is buffered. Ca^2+^ buffering proteins exist in the cytosol and bind to calcium ions, leaving a proportion of free unbound intracellular Ca^2+^ ions that can exert intracellular regulatory effects [22], [48]. The effects of two Ca^2+^ buffering proteins, calmodulin (CaM) and calreticulin (CRT) were included in the model [42]. An equation that describes the equilibrium of the buffering reactions was derived based on conservation of mass (provided in the Supporting Information S1) and was used to calculate the free intracellular Ca^2+^concentration. In addition, the intracellular Na^+^ and K^+^ concentrations were tracked over time by Eqs. 12 and 13:
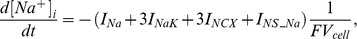
(12)

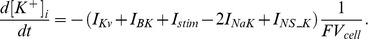
(13)


## Results

**Figure 8:**
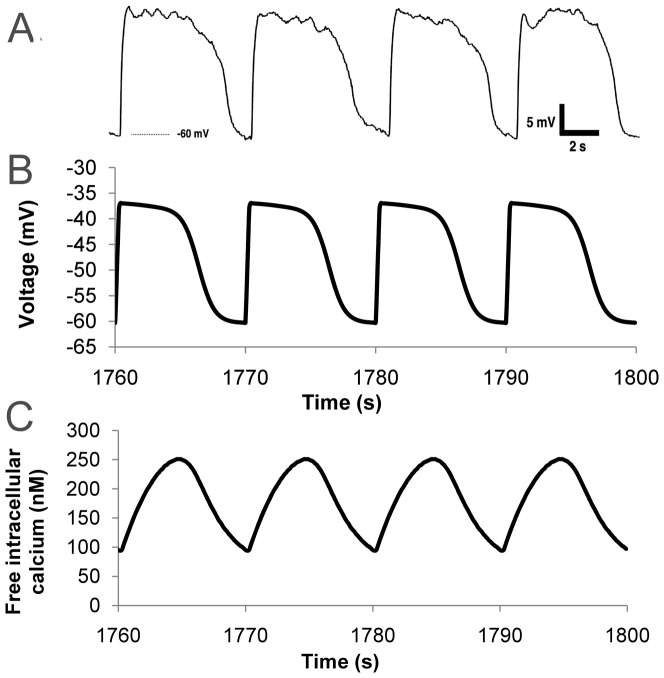
hJSMC simulation results. (a) Experimentally recorded hJSMC slow waves, reproduced from Lee et al [49] with permission. (b) Simulated hJSMC slow waves after a simulation of 30 minutes of electrical activity. (c) Predicted free intracellular calcium concentration.

Equation 1, which calculates 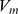 over time, the HH gating variables, and the ion concentrations were solved using an explicit forward Euler method. A time step of 0.1 ms was sufficient for convergence and stability. To solve the MM formulations with the same time step it was necessary to adopt a backward Euler approach. The predicted SMC slow wave profile, shown in Figure 8b, is in good quantitative agreement with experimental recordings from the human jejunum by Lee et al, shown in Figure 8a, with a peak amplitude of 23 mV and resting membrane potential of −60 mV [49]. Figure 8c shows the corresponding predicted free intracellular calcium concentration 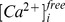 over time, which ranges from 94 nM at rest to 250 nM during the plateau phase. Experimental 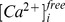 data for human intestinal smooth muscle is limited. Bielefeldt et al reported a 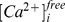 value of about 60 nM under resting conditions for the cultured human intestinal smooth muscle cells [50], while Farrugia et al reported a resting 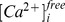 value of about 130 nM, and a maximum increase of about 160 nM [20]. Simulations were run for over 30 minutes of simulated time to ensure long term stability and ion homeostasis for the model.

**Figure 9:**
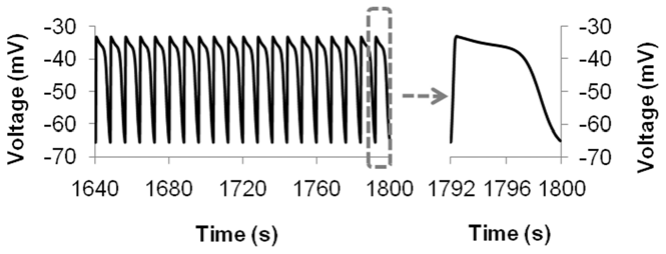
Simulated slow waves matched experimental control traces of **Figures 7A and 7B** in [**51**].

The human intestinal slow waves recorded in another study by Hwang et al [51] exhibited different properties from the results of Lee et al [49]. The average frequency was higher at 7.5 cpm (versus 6 cpm), and the amplitude was larger (31 mV) with a resting membrane potential of −64 mV. With an appropriately modified ICC stimulus, the hJSMC model was also able to reproduce the slow waves from Hwang et al as shown in Figure 9.

**Figure 10:**
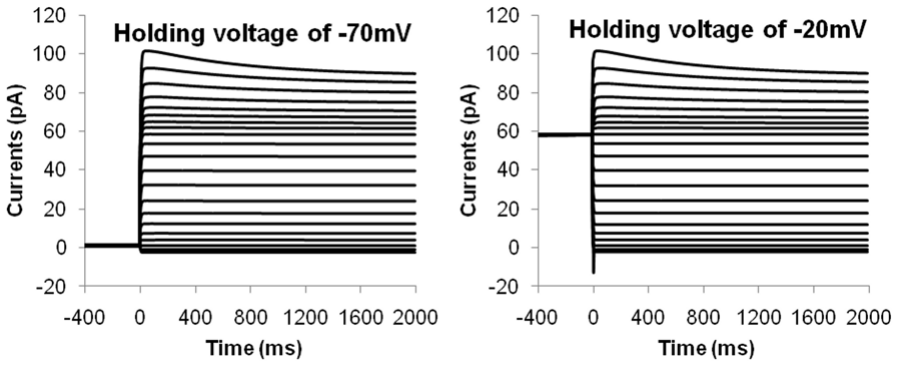
hJSMC whole cell current versus time traces subjected to voltage clamp at different holding voltages. The left figure shows the results for original holding voltage of −70 mV, while the right figure shows the results for a holding voltage of −20 mV.

Voltage clamp experiments on hJSMC revealed that the outward currents were not dependent on the holding voltage and did not inactivate significantly over a prolonged clamping period of 2000 ms [19]. The hJSMC model was subjected to equivalent voltage clamp conditions with clamping voltages ranging from −80 mV to 20 mV in increments of 5 mV. Figure 10 shows the simulation results of whole cell current versus time traces; the left panel is for a holding voltage of −70 mV while the right panel is for a holding voltage of −20 mV. This was repeated with other holding voltages from −90 mV to 0 mV. It was observed in these results that the steady-state behaviour of the whole cell currents is independent of holding voltage and has insignificant inactivation, which agrees with experimental observations [19].

**Figure 11:**
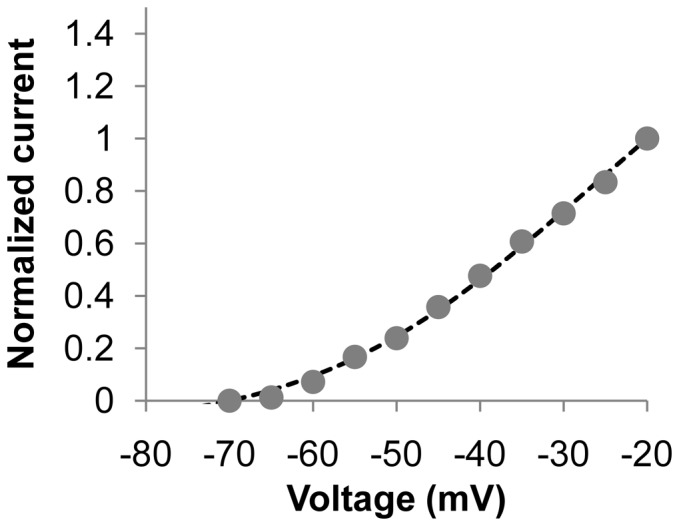
Predicted whole cell normalized I–V data from hJSMC model under near calcium-free conditions. The dashed line is the simulation data, while gray dots are experimental data [19] under control conditions.

In the same study, voltage clamp experiments on hJSMC in a Ca^2+^ free solution were performed and negligible changes in the I–V behaviour were found when compared to the original voltage clamp conditions [19]. To simulate this experiment, the Ca^2+^ concentrations were set to 0.0001 nM in the intracellular and extracellular spaces. The predicted I–V behaviour in Figure 11 (dashed line) is in good agreement the experimental results (gray dots).

**Figure 12:**
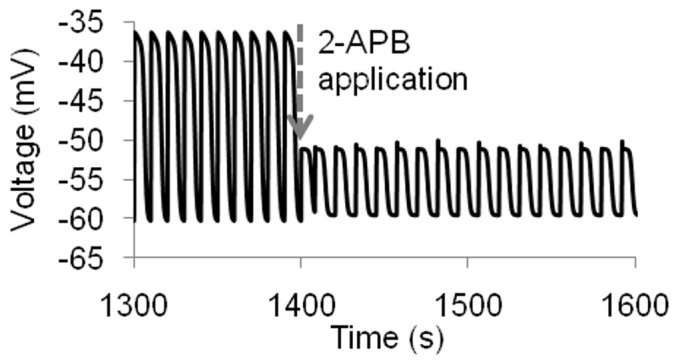
To simulate the effect of 2-APB, appropriate changes were made to the ICC stimulus. The stimulus was applied at a time point of 1400 s. Consequently, the hJSMC slow waves cycle at 4.9 times per minute, with a reduction in slow wave upstroke and plateau amplitude.

In the absence of a stimulus current from the ICC, the hJSMC model did not produce any spontaneous electrical activity. 2-Aminoetoxydiphenyl borate (2-APB) is a drug capable of inhibiting ICC slow waves; a concentration of 50 µM was able to reduce frequency to 4.90 cpm, increase time to peak by about 19.7% and reduce amplitude by about 32.9% [49]. The ICC stimulus was adjusted to reproduce this behaviour and Figure 12 shows the predicted change in hJSMC slow waves with the application of 2-APB.

## Discussion

This study proposes a model of human jejunal smooth muscle cell electrophysiology. To the best of our knowledge, this is the first biophysically based model of an intestinal SMC, and the first model of a human GI cell. The validation steps for the model were three-fold. First, the descriptions of the individual ionic currents were validated against the data from which they were constructed. Next, the hJSMC model was assembled and the resulting cellular behaviour was compared to whole cell experimental recordings. Finally, the response of the model under altered conditions (Ca^2+^ free, and with 2-APB) was checked.

**Figure 13:**
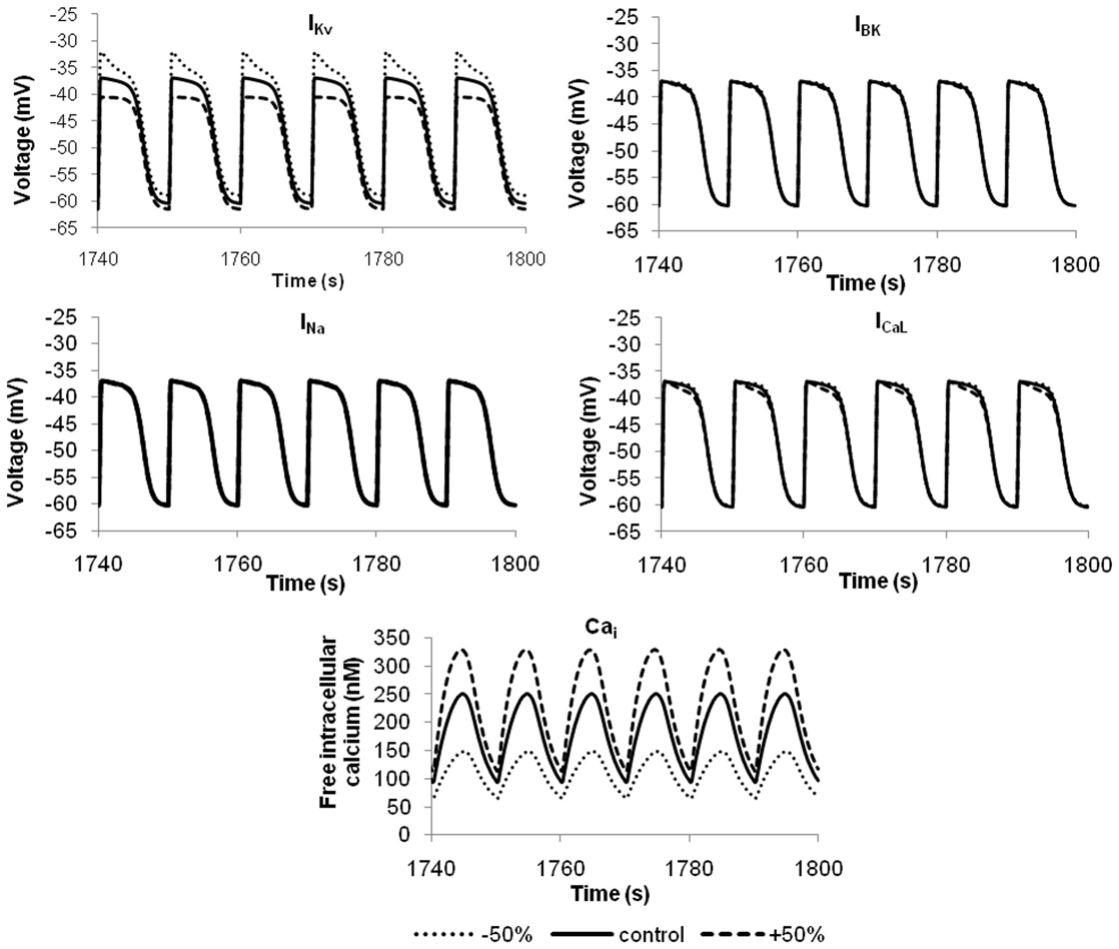
Sensitivity analysis by 50% increase or decrease in maximum channel conductance. This evaluates the contributions of key ionic currents towards hJSMC membrane voltage. (e) shows the free intracellular Ca^2+^ concentrations corresponding to changes in (d).

The resting membrane potential and the profile of the slow waves in hJSMC are determined by a balance of the ionic currents crossing the cell membrane. It is therefore useful to examine the contributions of the key currents that determine the shape of the SMC response to an ICC stimulus. Therefore, 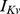, 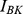, 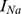 and 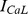 were individually subjected to a ±50% variation of their maximum conductances. It can be seen from Figure 13 that both the resting membrane potential and the slow wave plateau are most sensitive to 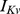. This is in agreement with experiments which demonstrated the voltage dependent K^+^ currents are dominant in shaping single cell slow waves [19]. Furthermore, a comparison of the I–V plots of other ionic currents (i.e., Figures 2, 3, 4, 5, 6) showed that the 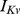 carries a relatively greater amplitude of current over the slow wave voltage range, and hence a greater influence on slow wave potentials. Additionally, it appears that 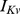 acts as a counter-balance to the stimulus current coming from the ICC. Interestingly, although alterations to the L-type Ca^2+^ current showed only minor differences in the plateau phase of the slow wave, those differences translated to significant differences in the amplitude of the intracellular Ca^2+^ transients. Neither 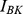 or 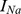 appeared to have a significant influence on the slow wave profile, but this observation must be tempered with the fact that here a prescribed ICC stimulus was used, meaning that the stimulus current from the ICC is in no way influenced by the electrical load placed on it by the SMC.

**Figure 14:**
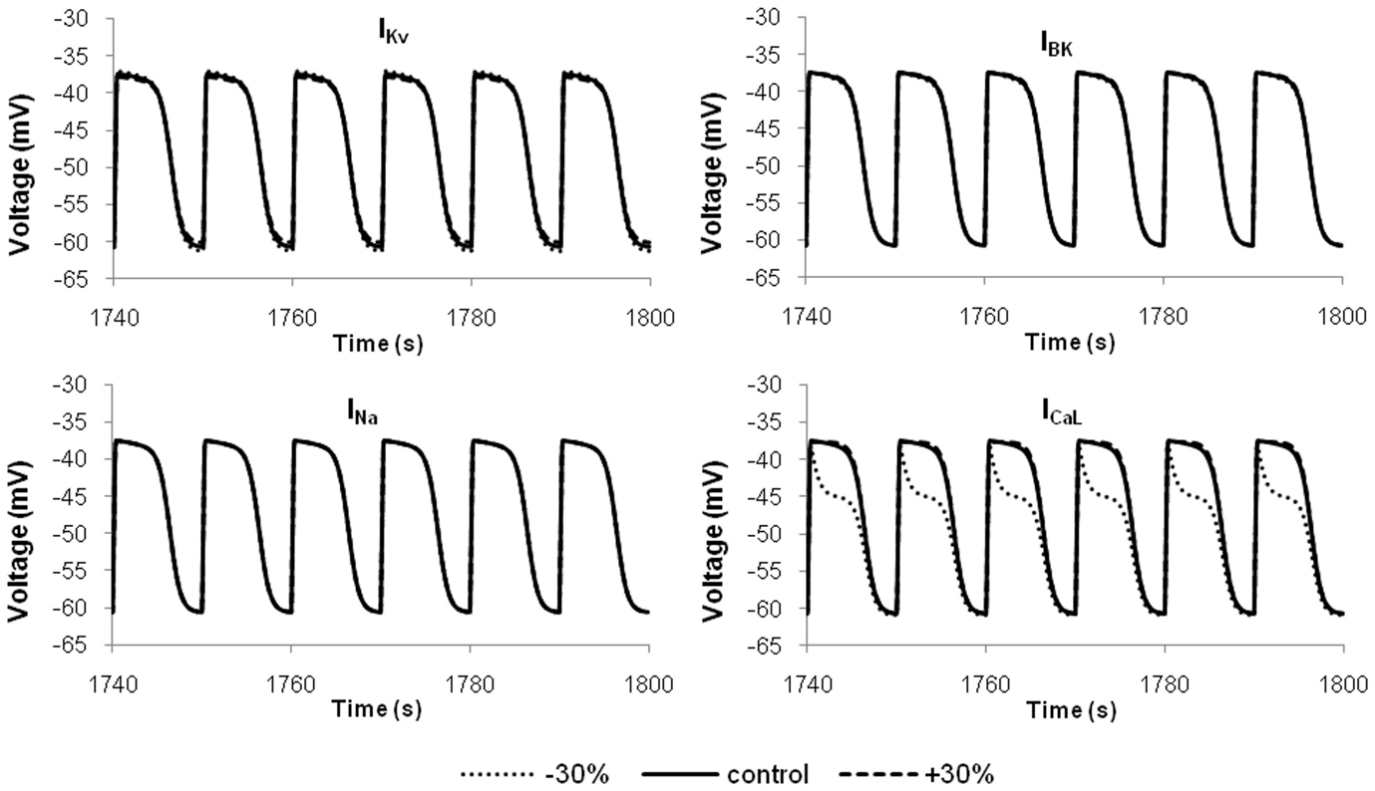
Selected results for further sensitivity analysis. The voltage-linked parameter values of channel activation for each of the four main ionic currents were varied by ±30%. From the simulation results, the hJSMC model remained robust with a preservation of general slow wave morphology.

Experimental data to obtain maximum conductance values are lacking, while more experimental data were available to parameterize the kinetic parameters of the main ionic conductances. Given the greater uncertainty in the maximum channel conductance values, the effects of ±50% variation in their values on cellular behaviour were examined, as described earlier. For the kinetic parameter values, further sensitivity analyses were performed for 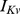, 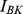, 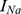 and 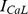 by using a ±30% change in the kinetic parameter values. The results demonstrated that the cellular slow waves were not visibly changed by changes in the inactivation parameters. For activation changes, cellular waves were noticeably altered for parameter variation in 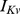 and 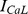 (as shown in Figure 14). The preservation of slow wave morphology in the results suggested the robustness of the model against parameter variations.

As some ionic currents present in the model were experimentally characterized in expression systems such as HEK cells, it was not possible to determine the maximum whole cell conductance from these experiments. In cases where whole cell data was available, this was used to determine the whole cell conductance. In cases where no appropriate whole cell data was found, the maximum conductance was carefully selected to satisfy what is known about those ionic currents and to reproduce the experimentally recorded hJSMC slow wave profiles.

The whole cell data used in the work was obtained from surgical waste tissue as this was the only human data available for model parameterization. These came from patients who underwent gastric bypass surgery as a treatment for morbid obesity. Studies on the smooth muscle tissue physiology for the obese are limited. A recent finding suggested enhanced human intestinal contractility in the obese due to increased sensitivity to excitatory neuronal modulation, but no significant change was observed with inhibitory neurotransmission tests [52]. Given the hJSMC model describes intrinsic electrophysiology, without neuronal modulation, the surgical waste tissue was therefore assumed not to limit the current model in describing the electrophysiology.

Another limitation in the model construction is the availability of experimental data from human jejunal cells. While the majority of the channels were constructed from human jejunal data, the BK channel model had to be parameterized from human myometrium smooth muscle data, while the homeostatic mechanisms were adopted from their human cardiac equivalents. As more relevant human jejunal data becomes available, the model can be updated accordingly.

The hJSMC mechanics is another important aspect that affects motility at the global level, with free intracellular calcium as a link between electrical and mechanical functions. With sufficient data, a mechanical hJSMC model can be developed and coupled to this electrical hJSMC model. Such a description can incorporate mechanosensitive ion channels to better examine their functional importance in the mechanically activated hJSMC [53], [54]. Finally, the equations and parameter values that define the hJSMC model are available in the Supporting Information S1. A sample implementation of the model is available upon request.

## Supporting Information

Supporting Information S1 (PDF)
