# Supplementary material for: A Quantitative Model of Human Jejunal Smooth Muscle Cell Electrophysiology
Source: PLoS One. 2012 Aug 17;7(8):e42385. doi: 10.1371/journal.pone.0042385 (PMC3422293; doi:10.1371/journal.pone.0042385)
Supplement: Supporting Information S1 — (PDF) [file pone.0042385.s001.pdf]

S1. SUPPORTING INFORMATION FOR THE HJSMC MODEL.

For the paper, "A quantitative model of human jejunal smooth muscle cell electrophysiology"

Yong Cheng Poh[a,b], Alberto Corrias[a], Nicholas Cheng[a], and Martin Lindsay Buist[a,b,*]

[a] Department of Bioengineering, National University of Singapore, 9 Engineering Drive 1, Block EA #03-12 Singapore 117576

[b] NUS Graduate School for Integrative Sciences and Engineering, National University of Singapore, Centre for Life Sciences (CeLS), #05-01 28 Medical Drive Singapore 117456

[*]To whom correspondence should be addressed: Faculty of Engineering, Block EA, #03-12, 9 Engineering Drive 1, Singapore 117576. Tel.: +(65) 6516-5929; Fax: +(65) 6872-3069; E-mail: biebml@nus.edu.sg.

Complete description of the hJSMC model:

The hJSMC model is described by Eqs. S-1 to S-96, and their parameter values are listed in Table S1.

Table S1. Model parameters

Parameter name	Description	Value	Units
R	Ideal gas constant	8.314	J/(molK)
F	Faraday's constant	96.48534	C/mmol
T	Temperature	310	K
C_m	Cell membrane capacitance	50	pF
V_{cell}	Cell volume	3.5e-12	l
$[Ca^{2+}]_o$	Extracellular calcium concentration	2	mM
$[K^+]_o$	Extracellular potassium concentration	5.4	mM
$[Na^+]_o$	Extracellular sodium concentration	140	mM
$[Ca^{2+}]_i^{total}$	Initial value of total intracellular calcium concentration	0.004914	mM
$[Ca^{2+}]_i^{free}$	Initial value of free intracellular calcium concentration	1.26e-4	mM
$[K^+]_i$	Intracellular potassium concentration	150	mM
$[Na^+]_i$	Intracellular sodium concentration	10.5	mM
Q_{10}^{Ca}	Q_{10} for calcium channels	2.1	-

Q_{10}^{K}	Q_{10} for potassium channels	3.1	-
Q_{10}^{Na}	Q_{10} for sodium channels	2.45	-
G_{couple}	Coupling conductance between ICC and SMC	2.6	nS
V_{rest}^{ICC}	Resting membrane potential of ICC	-57	mV
V_{peak}^{ICC}	Peak membrane potential of ICC	-23.5	mV
V_{amp}^{ICC}	Amplitude of ICC membrane potential (given by $V_{peak}^{ICC} - V_{rest}^{ICC}$)	33.5	mV
t_{period}	Period of single ICC slow wave	10000	ms
t_{peak}^{ICC}	Duration of ICC slow wave upstroke to reach V_{peak}^{ICC}	300	ms
$t_{plateau}^{ICC}$	Duration of ICC slow wave from start to plateau phase	9700	ms
t_{slope}	Slope factor in V_{m}^{ICC} equation	600	ms
f_1	ICC conditioning factor 1	12000	ms
f_2	ICC conditioning factor 2	300	ms
$[CRT]_{total}$	Total calreticulin concentration	0.034	mM
n_{CRT}	Hill coefficient for calreticulin	1	-
K_{D}^{CRT}	Dissociation constant for calreticulin	0.0009	mM
$[CaM]_{total}$	Total calmodulin concentration	0.012	mM
n_{CaM}	Hill coefficient for calmodulin	4	-
K_{D}^{CaM}	Dissociation constant for calmodulin	0.0001	mM^4
$\overline{G_{CaL}}$	Maximum conductance of I_{CaL}	1.44	nS
$\overline{G_{CaT}}$	Maximum conductance of I_{CaT}	0.0425	nS
$\overline{G_{Kv}}$	Maximum conductance of I_{Kv}	1.0217	nS
$\tau_{x_{Kv}}$	Time constant for x_{Kv} of I_{Kv}	4.7803	ms
$\tau_{y_{Kv}}$	Time constant for y_{Kv} of I_{Kv}	763.7564	ms
$\overline{G_{BK}}$	Maximum conductance of I_{BK}	80	nS
$\overline{G_{Na}}$	Maximum conductance of I_{Na}	25.1	nS
P_{NCX}	Maximum I_{NCX}	39.8437	pA/pF
K_{mCa}	$[Ca^{2+}]_i$ half saturation constant of I_{NCX}	1.38	mM
K_{mNai}	$[Na^{+}]_i$ half saturation constant of I_{NCX}	87.5	mM
k_{sat}	Saturation factor for I_{NCX}	0.1	-
γ	Voltage dependence parameter of I_{NCX}	0.35	-
P_{NaK}	Maximum I_{NaK}	0.1852	pA/pF
K_{mK}	$[K^{+}]_o$ half saturation constant of I_{NaK}	1	mM
K_{mNa}	$[Na^{+}]_i$ half saturation constant of I_{NaK}	40	mM

G_{NS_Na}	Maximum conductance of non-selective current carrying sodium ions, I_{NS_Na}	0.022488	nS
G_{NS_K}	Maximum conductance of non-selective current carrying potassium ions, I_{NS_K}	0.017512	nS

Complete equations of the hJSMC model

I. Governing equation for single hJSMC electrophysiology

Voltages in mV, ionic currents in pA

$$\frac{dV_m}{dt} = -\frac{I_{ion} + I_{Stim}}{C_m} \qquad \text{(S-1)}$$

II. Ionic currents, I_{ion}

$$I_{ion} = I_{CaL} + I_{CaT} + I_{Kv} + I_{BK} + I_{Na} + I_{NCX} + I_{NaK} + I_{NS} \qquad \text{(S-2)}$$

III. I_{Stim} equations

$$I_{Stim} = G_{couple}(V_m - V_m^{ICC}) \qquad \text{(S-3)}$$

Mathematical profile of the prescribed V_m^{ICC} that describes a single slow wave:

$$V_m^{ICC} = \begin{cases} V_{rest}^{ICC} + V_{amp}^{ICC}\left(\dfrac{t}{f_2}\right) & for \quad 0 \le t < t_{peak}^{ICC} \\ V_{rest}^{ICC} + V_{amp}^{ICC}\left(1+\exp\left(\dfrac{-f_1}{2t_{slope}}\right)\right)\left(\dfrac{1}{1+\exp\left(\dfrac{t-f_2-0.5f_1}{t_{slope}}\right)}\right) & for \quad t_{peak}^{ICC} \le t < t_{plateau}^{ICC} \end{cases} \qquad \text{(S-4)}$$

IV. Equations for tracking the intracellular ionic concentrations

Ion concentration should be tracked in mM

$$\frac{d[Ca^{2+}]_i^{total}}{dt} = -\left(I_{CaL} + I_{CaT} - 2I_{NCX}\right)\frac{1}{2FV_{cell}} \tag{S-5}$$

$$\frac{d[Na^{+}]_i}{dt} = -\left(I_{Na} + 3I_{NaK} + 3I_{NCX} + I_{NS\_Na}\right)\frac{1}{FV_{cell}} \tag{S-6}$$

$$\frac{d[K^{+}]_i}{dt} = -\left(I_{Kv} + I_{BK} + I_{stim} - 2I_{NaK} + I_{NS\_K}\right)\frac{1}{FV_{cell}} \tag{S-7}$$

V. Nernst potential

Nernst potential unit is mV

$$E_{Ca} = \frac{RT}{2F}\ln\frac{[Ca^{2+}]_o}{[Ca^{2+}]_i} \tag{S-8}$$

$$E_{K} = \frac{RT}{F}\ln\frac{[K^{+}]_o}{[K^{+}]_i} \tag{S-9}$$

$$E_{Na} = \frac{RT}{F}\ln\frac{[Na^{+}]_o}{[Na^{+}]_i} \tag{S-10}$$

VI. Calcium buffering

Calcium concentration in mM

$$\frac{d[Ca^{2+}]_i^{free}}{dt} = \frac{d[Ca^{2+}]_i^{total}}{dt} \div \left(1 + \frac{n_{CRT}[CRT]_{total}K_D^{CRT}\left([Ca^{2+}]_i^{free}\right)^{n_{CRT}-1}}{\left(\left([Ca^{2+}]_i^{free}\right)^{n_{CRT}} + K_D^{CRT}\right)^2} + \frac{n_{CaM}[CaM]_{total}K_D^{CaM}\left([Ca^{2+}]_i^{free}\right)^{n_{CaM}-1}}{\left(\left([Ca^{2+}]_i^{free}\right)^{n_{CaM}} + K_D^{CaM}\right)^2}\right) \tag{S-11}$$

VII. L-type calcium current, I_{CaL}

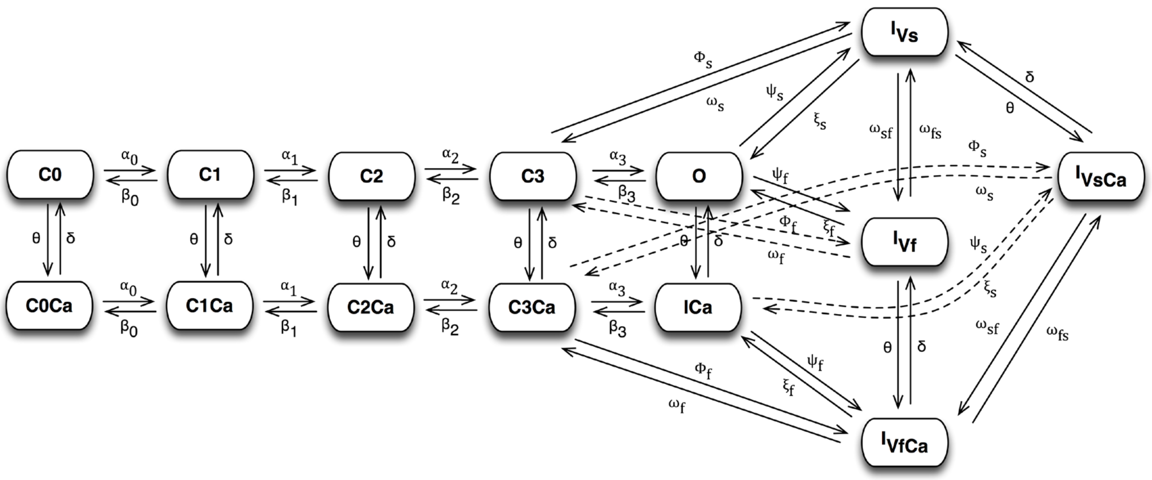

Figure S1. L-type Ca^{2+} channel Markov model topology. Prefixes *C, I,* O denote closed, inactivated and open states respectively; suffixes *Ca, V_m, f,* s found in some of the states refer to calcium-bound, voltage-dependent, fast, and slow properties of these states respectively. The topology was designed to best describe observed characteristics of the L-type Ca^{2+} channels. O is the state that conducts Ca^{2+} ions across the channels.

$$I_{CaL} = \overline{G_{CaL}} P_O (V_m - E_{Ca}) \tag{S-12}$$

Common rate equations:

$$a = 0.7310 \exp(\frac{V_m}{30.0}) \tag{S-13}$$

$$b = 0.2149 \exp(\frac{-V_m}{40.0}) \tag{S-14}$$

Rate equations for horizontal activation transitions:

$$a_0 = 4a \tag{S-15}$$

$$a_1 = 3a \tag{S-16}$$

$$a_2 = 2a \tag{S-17}$$

$$a_3 = a \tag{S-18}$$

Rate equations for horizontal deactivation transitions (ms^{-1}):

$$b_0 = b \tag{S-19}$$

$$b_1 = 2b \tag{S-20}$$

$$b_2 = 3b \tag{S-21}$$

$$b_3 = 4b \tag{S-22}$$

Rate equations for fast and slow inactivation transitions (ms^{-1}):

$$\phi_f = 0.4742\exp(\frac{V_m}{10.0}) \tag{S-23}$$

$$\phi_s = 0.05956\exp(\frac{-V_m}{40.0}) \tag{S-24}$$

$$\xi_f = 0.01407\exp(\frac{-V_m}{300.0}) \tag{S-25}$$

$$\xi_s = 0.01213\exp(\frac{V_m}{500.0}) \tag{S-26}$$

$$\psi_f = 0.02197\exp(\frac{V_m}{500.0}) \tag{S-27}$$

$$\psi_s = 0.00232\exp(\frac{-V_m}{280.0}) \tag{S-28}$$

$$\omega_f = \frac{b_3 \xi_f \phi_f}{a_3 \psi_f} \tag{S-29}$$

$$\omega_s = \frac{b_3 \xi_s \phi_s}{a_3 \psi_s} \tag{S-30}$$

$$\omega_{sf} = \frac{\xi_s \psi_f}{\xi_f} \tag{S-31}$$

$$\omega_{fs} = \psi_s \tag{S-32}$$

Rate equations for calcium dependent transitions (ms^{-1}):

$$\theta = \frac{4}{1 + \frac{1}{[Ca^{2+}]_i^{free}}} \tag{S-33}$$

$$\delta = 0.01 \tag{S-34}$$

VIII. T-type calcium current, I_{CaT}

$$I_{CaT} = \overline{G_{CaT}} d_{CaT} f_{CaT} (V_m - E_{Ca}) \tag{S-35}$$

Equations for gating variables (ms^{-1}):

$$\frac{dd_{CaT}}{dt} = \frac{d_{CaT}^{\infty} - d_{CaT}}{\tau_{d_{CaT}}} \tag{S-36}$$

$$\frac{df_{CaT}}{dt} = \frac{f_{CaT}^{\infty} - f_{CaT}}{\tau_{f_{CaT}}} \tag{S-37}$$

Equations for steady-state values of the gating variables:

$$d_{CaT}^{\infty} = \frac{1}{1 + \exp\left(-\frac{V_m + 60.5}{5.3}\right)} \tag{S-38}$$

$$f_{CaT}^{\infty} = \frac{1}{1 + \exp\left(\frac{V_m + 75.5}{4.0}\right)} \tag{S-39}$$

Equations for the time constant variables:

$$\tau_{d_{CaT}} = 1.9058 \quad \text{(S-40)}$$

$$\tau_{f_{CaT}} = 0.38117\left(8.6 + 14.7\exp\left(-\frac{(V_m + 50)^2}{900}\right)\right) \quad \text{(S-41)}$$

IX. Voltage dependent potassium current, I_{Kv}

$$I_{Kv} = \overline{G_{Kv}} x_{Kv} y_{Kv} (V_m - E_K) \quad \text{(S-42)}$$

Rate equations for the gating variables (ms^{-1}):

$$\frac{dx_{Kv}}{dt} = \frac{x_{Kv}^{\infty} - x_{Kv}}{\tau_{x_{Kv}}} \quad \text{(S-43)}$$

$$\frac{dy_{Kv}}{dt} = \frac{y_{Kv}^{\infty} - y_{Kv}}{\tau_{y_{Kv}}} \quad \text{(S-44)}$$

Equations for the steady-state values of the gating variables:

$$x_{Kv}^{\infty} = \frac{1}{1 + \exp\left(-\frac{V_m + 43.0}{17.36}\right)} \quad \text{(S-45)}$$

$$y_{Kv}^{\infty} = \frac{1}{1 + \exp\left(\frac{V_m - 44.9}{12.0096}\right)} \quad \text{(S-46)}$$

X. Calcium & voltage activated potassium current, I_{BK}

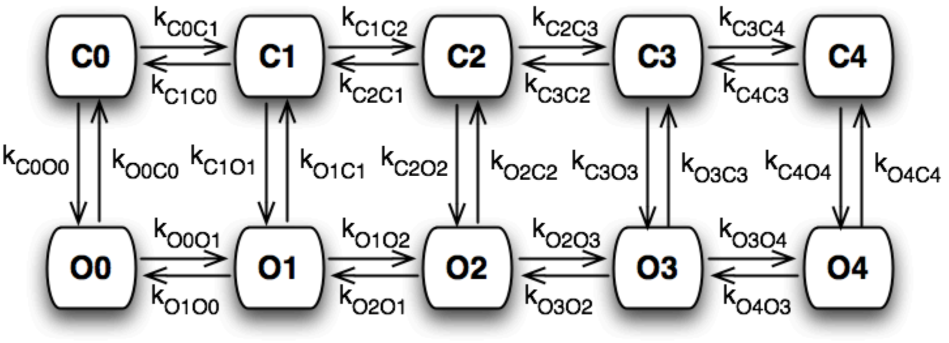

Figure S2. A10-state Markov model of homotetrameric BK channel. Upper tier states are various closed conformation states (with prefix *C*) while lower tier states are various open-oriented conformation states (with prefix *O*). In each tier, the horizontal transitions depend on $[Ca^{2+}]_i^{free}$, that reflects cooperative Ca^{2+} binding to each of the four alpha subunits of the BK homotetramer; the membrane voltage dependency is found in the vertical transitions between states. O4 is the conformation state that conducts ions across BK channels under a voltage driving force.

$$I_{BK} = \overline{G_{BK}} P_O (V_m - E_K) \quad \text{(S-47)}$$

Common rate equations:

$$a = \exp(\frac{8.47188 V_m}{T}) \quad \text{(S-48)}$$

$$b = \exp(\frac{-7.77556 V_m}{T}) \quad \text{(S-49)}$$

$$k_{on} = 40633 \quad \text{(S-50)}$$

$$k_{off}^{C} = 11 \quad \text{(S-51)}$$

$$k_{off}^{O} = 1.1 \quad \text{(S-52)}$$

Rate equations for voltage dependent transitions (ms^{-1}):

$$k_{C0O0} = 0.02162a \quad \text{(S-53)}$$

$$k_{C1O1} = 0.000869a \quad \text{(S-54)}$$

$$k_{C2O2} = 0.0000281a \tag{S-55}$$

$$k_{C3O3} = 0.000781a \tag{S-56}$$

$$k_{C4O4} = 0.044324a \tag{S-57}$$

$$k_{O0C0} = 318.1084b \tag{S-58}$$

$$k_{O1C1} = 144.1736b \tag{S-59}$$

$$k_{O2C2} = 32.6594b \tag{S-60}$$

$$k_{O3C3} = 0.095312b \tag{S-61}$$

$$k_{O4C4} = 0.000106b \tag{S-62}$$

Rate equations for calcium dependent transitions (ms^{-1}):

$$k_{C0C1} = 4k_{on}[Ca^{2+}]_i^{free} \tag{S-63}$$

$$k_{C1C2} = 3k_{on}[Ca^{2+}]_i^{free} \tag{S-64}$$

$$k_{C2C3} = 2k_{on}[Ca^{2+}]_i^{free} \tag{S-65}$$

$$k_{C3C4} = k_{on}[Ca^{2+}]_i^{free} \tag{S-66}$$

$$k_{C4C3} = 4k_{off}^{C}[Ca^{2+}]_i^{free} \tag{S-67}$$

$$k_{C3C2} = 3k_{off}^{C}[Ca^{2+}]_i^{free} \tag{S-68}$$

$$k_{C2C1} = 2k_{off}^{C}[Ca^{2+}]_i^{free} \tag{S-69}$$

$$k_{C1C0} = k_{off}^{C}[Ca^{2+}]_i^{free} \tag{S-70}$$

$$k_{O0O1} = 4k_{on}[Ca^{2+}]_i^{free} \tag{S-71}$$

$$k_{O1O2} = 3k_{on}[Ca^{2+}]_i^{free} \tag{S-72}$$

$$k_{O2O3} = 2k_{on}[Ca^{2+}]_i^{free} \tag{S-73}$$

$$k_{O3O4} = k_{on}[Ca^{2+}]_i^{free} \tag{S-74}$$

$$k_{O4O3} = 4k_{off}^{O}[Ca^{2+}]_i^{free} \tag{S-75}$$

$$k_{O3O2} = 3k_{off}^{O}[Ca^{2+}]_i^{free} \tag{S-76}$$

$$k_{O2O1} = 2k_{off}^{O}[Ca^{2+}]_i^{free} \tag{S-77}$$

$$k_{O1O0} = k_{off}^{O}[Ca^{2+}]_i^{free} \tag{S-78}$$

XI. Voltage dependent sodium current, I_{Na}

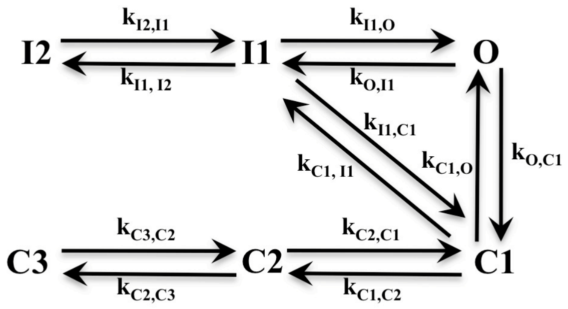

Figure S3. Six-state Markov model of $Na_V1.5$. *C* refers to closed state, *I* refers to inactivated state, and *O* refers to open state where Na^+ ions are conducted across the channels.

$$I_{Na} = \overline{G_{Na}} P_O (V_m - E_{Na}) \tag{S-79}$$

Rate equations (ms^{-1}):

$$k_{O,I1} = 1.6164 \exp(0.30763 + 0.0060535 V_m) \tag{S-80}$$

$$k_{I1,I2} = 0.027735 \exp(0.051490 - 0.046865 V_m) \tag{S-81}$$

$$k_{C3,C2} = 0.00052548 \exp(-0.069102 + 0.0031945 V_m) \tag{S-82}$$

$$k_{C2,C1} = 1.4496 \exp(-0.15660 + 0.058353 V_m) \tag{S-83}$$

$$k_{C1,O} = 1.5329 \exp(0.0093193 + 0.041075 V_m) \tag{S-84}$$

$$k_{I2,I1} = 0.0039239 \exp(2.6793 + 0.0061468 V_m) \tag{S-85}$$

$$k_{C2,C3} = 0.55432\exp(-0.099074 + 0.036441V_m) \quad \text{(S-86)}$$

$$k_{C1,C2} = 3.1566\exp(0.36352 + 0.077193V_m) \quad \text{(S-87)}$$

$$k_{O,C1} = 2.3915\exp(-13.335 - 0.25289V_m) \quad \text{(S-88)}$$

$$k_{I1,C1} = 1.9046\exp(-2.4840 + 0.020406V_m) \quad \text{(S-89)}$$

$$k_{C1,I1} = 0.00021688\exp(-0.063438 + 0.0046683V_m) \quad \text{(S-90)}$$

$$k_{I1,O} = 0.12052\exp(-9.6028 + 0.083025V_m) \quad \text{(S-91)}$$

XII. Sodium calcium exchanger, I_{NCX}

$$I_{NCX} = P_{NCX}\frac{\exp\left(\frac{\gamma V_m F}{RT}\right)[Na^+]_i^3[Ca^{2+}]_o - 2.5\exp\left(\frac{(\gamma - 1.0)V_m F}{RT}\right)[Na^+]_o^3[Ca^{2+}]_i^{free}}{\left(K_{mNai}{}^3 + [Na^+]_o{}^3\right)\left(K_{mCa} + [Ca^{2+}]_o\right)\left(1 + k_{sat}\exp\left(\frac{(\gamma - 1.0)V_m F}{RT}\right)\right)} \quad \text{(S-92)}$$

XIII. Sodium potassium pump, I_{NaK}

$$I_{NaK} = P_{NaK}\frac{K_O[Na^+]_i}{([K^+]_o + K_{mK})([Na^+]_i + K_{mNa})\left(1 + 0.1245\exp\left(-\frac{0.1V_m F}{RT}\right) + 0.0353\exp\left(-\frac{V_m F}{RT}\right)\right)} \quad \text{(S-93)}$$

XIV. Non selective leak current

$$I_{NS} = I_{NS\_Na} + I_{NS\_K} \quad \text{(S-94)}$$

$$I_{NS\_Na} = g_{NS\_Na}\left(V_m - E_{Na}\right) \quad \text{(S-95)}$$

$$I_{NS\_K} = g_{NS\_K}\left(V_m - E_K\right) \quad \text{(S-96)}$$

Detailed simulated patch clamp experimental data:

The current versus voltage (I-V) plots of the ionic conductances shown in the manuscript, were obtained from the detailed current over time data from patch clamp simulations. These simulations followed the experimental protocol from their original papers. These data were chosen because the experimental patch clamp measured ion channel behaviour that covered the physiological range of membrane voltages and calcium concentrations. Therefore, the experimental data were deemed suitable to create ion channel models that were integrated to the single cell framework. This section shows the detailed simulation data for each of the ionic conductances accompanied by a brief description of the patch clamp conditions.

I_{CaL}

The patch clamp protocol has a holding voltage of -100 mV, and a clamping voltage range of -90 mV to 30 mV with a step size of 10 mV. Clamping voltage duration is 40 ms, while start to start time is 1 s [25]. The simulated L-type calcium current over time results are shown in figure S4, while the protocol is shown in the inset.

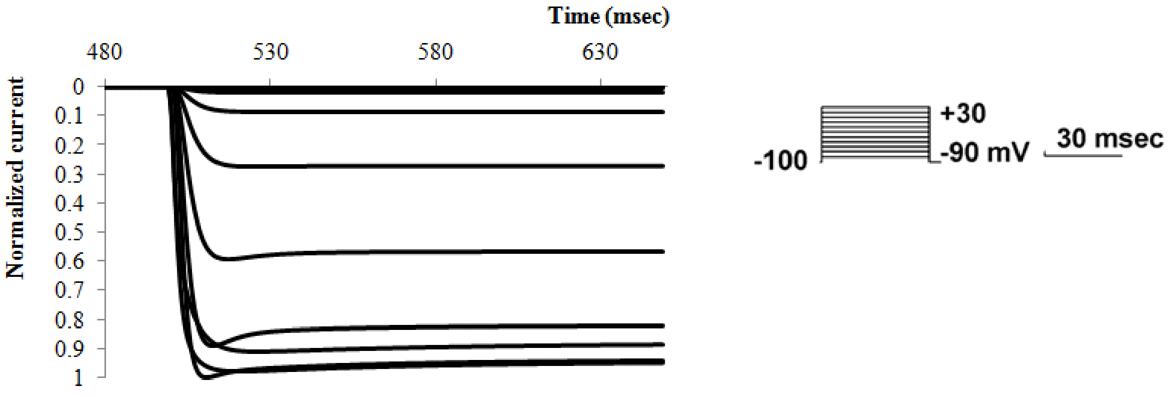

Figure S4. Current over time simulation results for I_{CaL}. Inset shows the voltage clamp protocol, and its corresponding time scale.

I_{CaT}

The standard activation patch clamp protocol was used where the holding voltage is -100 mV, and the clamping voltage range is from -90 mV to 35 mV with a step size of 5 mV. Clamping voltage duration is 400 ms, while start to start time is 1 s [28]. The simulated T-type calcium current over time results are shown in figure S5.

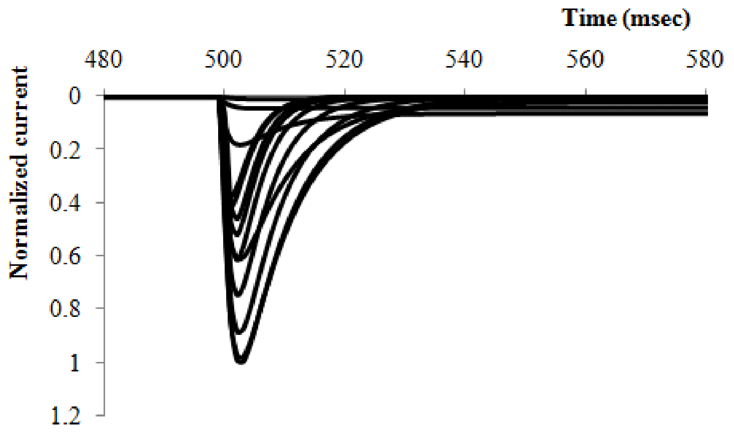

Figure S5. Current over time simulation results for I_{CaT}.

I_{Kv}

The patch clamp protocol has a holding voltage of -70 mV, and a clamping voltage range of -90 mV to 45 mV. Clamping voltage duration is 180 ms, while start to start time is assumed to be 1 s [29]. The simulated potassium current over time results are shown in figure S6, while the protocol is shown in the inset.

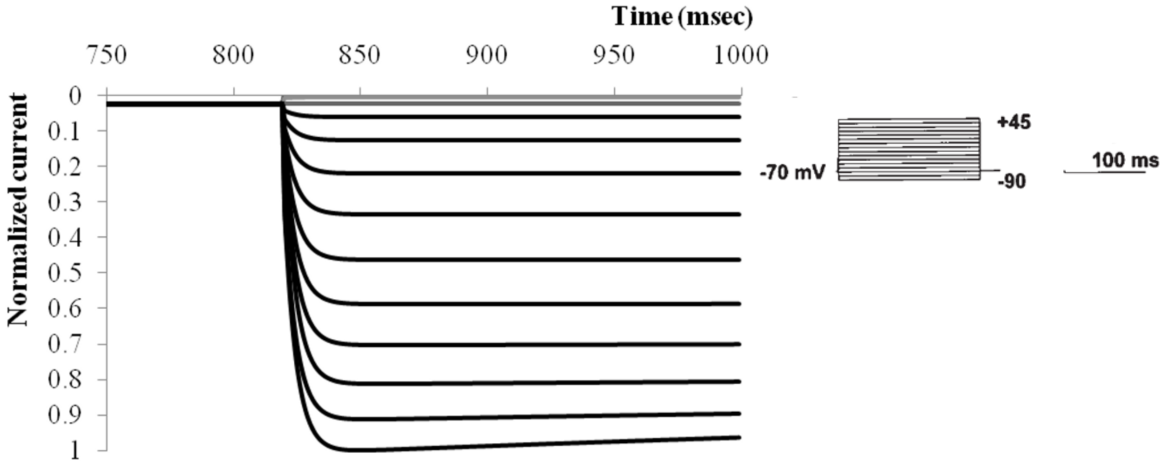

Figure S6. Current over time simulation results for I_{Kv}. Inset shows the voltage clamp protocol, and its corresponding time scale.

I_{BK}

The standard activation patch clamp protocol was applied over three intracellular calcium concentrations of 100 nM, 300 nM, and 1000 nM. For each concentration, the clamping voltage range is from -70 mV to 60 mV. The simulated open probability over time results, for each of the calcium concentrations, are shown in figure S7.

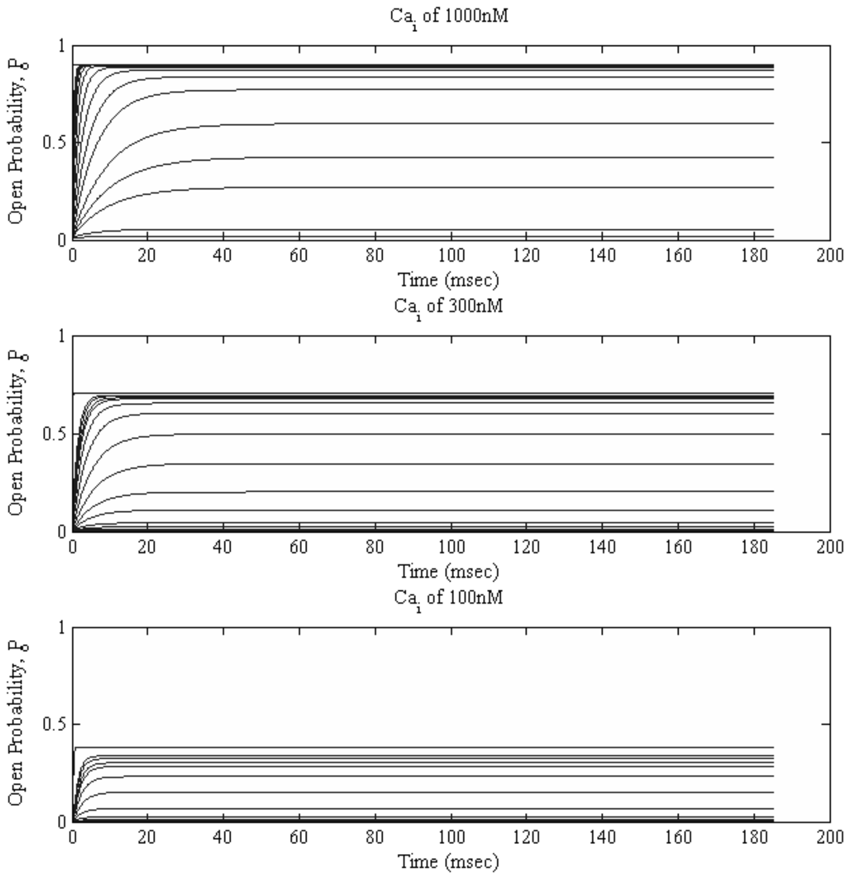

Figure S7. Current over time simulation results for I_{BK}. The subplots correspond to three different calcium concentrations of 100 nM, 300 nM and 1000 nM.

I_{Na}

The patch clamp protocol has a holding voltage of -100 mV, and a clamping voltage range of -80 mV to 35 mV with a step size of 5 mV. Clamping voltage duration is 50 ms, while start to start time is 1 s [36]. The simulated sodium current over time results are shown in figure S8, while the protocol is shown in the inset.

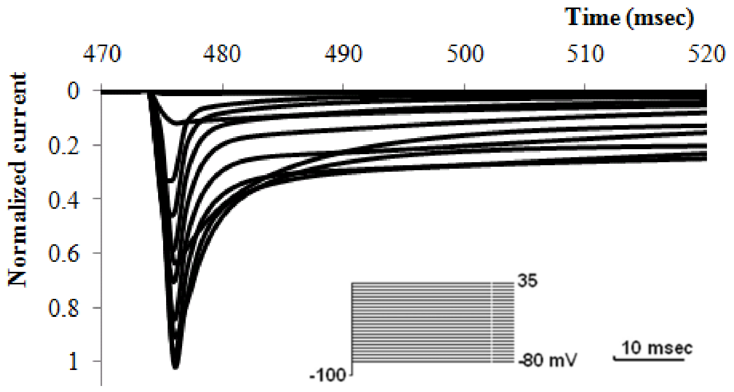

Figure S8. Current over time simulation results for I_{Na}. Inset shows the voltage clamp protocol, and its corresponding time scale.
